# Cytotoxic Phosphinoarsino and Diphosphino Pd(II) Complexes of Thiolate Amino Acids and Glutathione

**DOI:** 10.1155/MBD.1995.19

**Published:** 1995

**Authors:** Orla M. Ni Dhubhghaill, Peter J. Sadler, Esther Garcia Fernandez

**Affiliations:** 1 Department of Chemistry Birkbeck College University of London Gordon House, 29 Gordon Square London WC1H OPP United Kingdom; 2 Department of Inorganic Chemistry University of Santiago de Compostela Campus Universitario Santiago de Compostela E-15706 Spain

## Abstract

The novel complexes [Pd(L-L′)(SR)Cl] where L-L′ is Ph_2_PCH_2_CH_2_PPh_2_ (dppe) or Ph_2_AsCH_2_CH_2_PPh_2_ (dadpe) and RSH is glutathione, L-cysteine, or N-acetyl-L-cysteine, have been prepared and characterised. Their structures in the solid-state and in solution are discussed. The introduction of cysteine or glutathione as a ligand in these complexes greatly improved their aqueous solubility compared with the hydrophobic parent dichloro complexes. The cytotoxicities of the glutathione complexes towards the cell-lines L1210, ADJ/PC6 and CH1 were investigated. Their cytotoxicities towards L1210 cells were comparable to those of the parent dichloro complexes.


### CYTOTOXIC PHOSPHINOARSINO AND DIPHOSPHINO Pd(II) COMPLEXES OF THIOLATE AMINO ACIDS AND GLUTATHIONE

Orla M. Ni Dhubhghaill[1], Peter J. Sadler[1] and Esther Garcia Fernandez[2]

[1] Department of Chemistry, Birkbeck College, University of London, Gordon House, 29 Gordon Square, London WC1H OPP, United Kingdom
[2] Department of Inorganic Chemistry, University of Santiago de Compostela, Campus Universitario, E-15706 Santiago de Compostela, Spain

### ABSTRACT

The novel complexes [Pd(L-L′)(SR)Cl] where L-L′ is $Ph_2PCH_2CH_2PPh_2$ (dppe) or $Ph_2AsCH_2CH_2PPh_2$ (dadpe) and RSH is glutathione, L-cysteine, or N-acetyl-L-cysteine, have been prepared and characterised. Their structures in the solid-state and in solution are discussed. The introduction of cysteine or glutathione as a ligand in these complexes greatly improved their aqueous solubility compared with the hydrophobic parent dichloro complexes. The cytotoxicities of the glutathione complexes towards the cell-lines L1210, ADJ/PC6 and CH1 were investigated. Their cytotoxicities towards L1210 cells were comparable to those of the parent dichloro complexes.

### ABBREVIATIONS

dppe 1,2-diphenylphosphinoethane; dadpe 1-diphenylarsino-2-diphenylphosphinoethane

$H_2$dmsa dimercaptosuccinic acid; GSH glutathione, γ-L-Glu-L-Cys-Gly

CysH L-cysteine; N-AcCysH N-acetyl-L-cysteine

$IC_{50}$ concentration required to kill 50% of the cells

### INTRODUCTION

Certain tetrahedral diphosphine complexes of Group 11 metals, such as $[Au(dppe)_2]Cl$ are highly cytotoxic and exhibit anticancer activity in some model systems.[1] They appear to act by a different mechanism to cisplatin, *cis*-$[PtCl_2(NH_3)_2]$, attacking proteins as well as DNA. Recently we have sought to extend this series of complexes to include square-planar diphosphine complexes.[2] One of the major objectives is to increase the aqueous solubility of complexes containing hydrophobic diphosphine ligands, and in this paper we use thiolate amino acid derivatives for this purpose.

The formation of Pd(II) complexes with amino acids and peptides has been reviewed by Pettit and Bezer.[3] There have been few studies on the formation of Pd(II) complexes with glutathione (γ-L-Glu-L-Cys-Gly),[3,4,5] although there are some reports of $^1H$ NMR studies on binding of GSH to other metals, eg $Me_3Pb$,[6] mixed ligand complexes of Cd(II)(NTA),[7] and gold (I) drugs.[8] Rabenstein and coworkers have carried out $^1H$ and $^{13}C$ NMR spectroscopic studies on the binding[9], formation constants,[10] and ligand exchange reactions[11] of MeHg(II)-thiol complexes with thiols such as glutathione, cysteine. There are no *X*-ray structures of metal GSH complexes, however a dimeric Cu(II) complex of GSSG has been crystallographically characterised.[12] The solid state structure of the complex of Pd(II) with S-methyl-L-cysteine has been reported.[13] The geometry about the metal is square planar with S, N coordination , as has been found for Pd complexes of S-methyl-L-cysteine sulphoxide[14] and S-methyl-L-cysteine methyl ester. [15]

We report here the preparation and characterization of novel mixed ligand Pd(II) complexes containing cysteine, N-acetylcysteine or glutathione, and diphosphine or arsinophosphines as ligands, and their cytotoxicity against L1210, ADJ/PC6 and CH1 cell-lines.

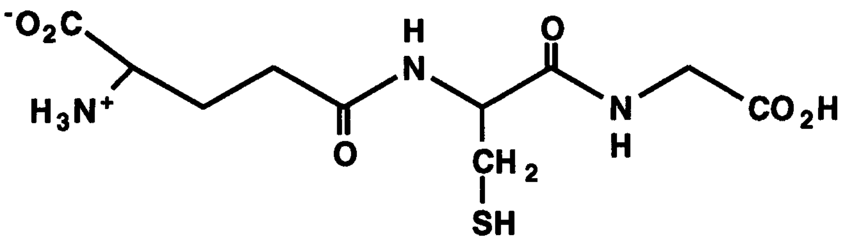

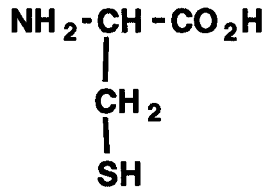

**Glutathione (GSH): γ-L-Glu-L-Cys-Gly** **L-Cys**

### EXPERIMENTAL SECTION

1,2-Bis(diphenylphosphino)-ethane (dppe) and 1-diphenylphosphino-2-diphenylarsinoethane (dadpe) were purchased from Strem Chemicals. L-Cysteine (free base), N-acetyl-L-cysteine, and glutathione were obtained from Sigma. The compounds [Pd(L-L′)$Cl_2$] (L-L′ = dppe, dadpe) were prepared from dichloro-1,5-cyclooctadiene-palladium(II) (Aldrich) as previously described for the analogous Pt complex,[16] and isolated as $CHCl_3$ solvates, [Pd(L-L′)$Cl_2$].2$CHCl_3$.

### Physical measurements

500 MHz $^1$H NMR spectra were recorded at ambient temperature (*ca.* 23°C) on a JEOL GSX500 instrument. $^{13}$C{$^1$H} NMR spectra were recorded on a JEOL GSX270 spectrometer operating at 71.9 MHz; 81 MHz $^{31}$P{$^1$H} spectra were acquired on a Bruker WM200 spectrometer. $^1$H and $^{13}$C chemical shifts were referenced to sodium trimethylsilyl-2,2,3,3-$d_4$-propionate (TSP), and $^{31}$P shifts to external 85% $H_3PO_4$.

Measurements of pH* (pH meter reading in $D_2O$ solutions) were made on a Corning pH meter equipped with an Aldrich combination microelectrode. Adjustments of pH* were made with DCl or NaOD.

IR spectra were recorded on a Perkin-Elmer 1330 instrument as Nujol mulls for GSH and its complexes, and [Pd(dadpe)(NAcCys)Cl], and as KBr discs for CysH and [Pd(dppe)(Cys)Cl]. For

[Pd(dadpe)(Cys)Cl] a KBr disc was used between 4000 and 600 $cm^{-1}$ and a Nujol mull between 600 and 200 $cm^{-1}$.

Mass spectra (fast atom bombardment) were recorded using a VG ZAB-SE mass spectrometer at the University of London School of Pharmacy (ULIRS) with samples in an MNOBA (meta-nitro-ortho-benzoic acid) matrix. Elemental analyses were carried out by the microanalysis service at University College London.

Thin layer chromatography was carried out on RPS-F plates (Anachem) using 1:1 EtOH:$H_2O$ as eluant. Cysteine complexes were dissolved in EtOH or $H_2O$ prior to elution and glutathione complexes in $H_2O$.

**Synthesis of [Pd(dppe)(Cys.HCl)Cl].$H_2O$ (1)**

To a suspension of [Pd(dppe)$Cl_2$].2$CHCl_3$ (0.099 g, 0.12 mmol) in aq. EtOH (30 mL, 1:2) was added an aqueous solution (5 mL) of L-CysH (0.014 g, 0.12 mmol). After stirring at room temperature for 1 h, the solution had become yellow with some solid present. It was filtered, then concentrated affording a *yellow* solid **1** (0.0145 g, 17%), m.p. 170 $^{o}C$.

(Found: C, 47.0; H, 4.6; N, 2.2; P, 9.1 %; $C_{29}H_{33}NO_3SP_2Cl_2Pd$ requires : C, 48.7; H, 4.65; N, 2.0; P, 8.7 %); IR data, Table 1; $^{31}P$ and $^{1}H$ NMR data, Tables 2 and 3; *m/z* 624 {[Pd(dppe)Cys]$^+$}. Tlc: $R_f$ = 0.64 EtOH solution; $R_f$ = 0.58 and faint spot at $R_f$ = 0.83 aq. solution)

**Synthesis of [Pd(dadpe)(Cys.HCl)Cl ] .$H_2O$ (2)**

To a suspension of [Pd(dadpe)$Cl_2$].2$CHCl_3$ (0.202 g, 0.235 mmol) in aq. EtOH (30 mL, 1:2) was added L-CysH (0.028 g, 0.23 mmole) in 5 mL $H_2O$. After stirring at room temperature for 1 h, the almost clear yellow solution was filtered and the filtrate concentrated affording an orange-yellow solid **2**. (0.137 g, 77%).

(Found: C, 45.5; H, 4.3; N, 1.7; P, 4.6 %; $C_{29}H_{33}NO_3SPAsCl_2Pd$ requires: C, 45.9 ; H, 4.4; N,

1.85; P, 4.1 %); IR data, Table 1; $^{31}$P NMR data, Table 2; *m/z* 670 {[Pd(dadpe)(CysH)]$^+$ + 1}. Tlc :$R_f$ = 0.71 and 0.69 for EtOH and $H_2O$ solutions, respectively)

**Synthesis of [Pd(dppe)(GS.HCl)Cl].3$H_2O$ (3)**

To a suspension of [Pd(dppe)$Cl_2$].2$CHCl_3$ (0.166 g, 0.203 mmol) in aq. EtOH (30 mL, 1:2) was added an aqueous solution (10 mL) of GSH (0.071 g, 0.229 mmol). After stirring at room temperature for 1 h, the solution had become almost clear yellow. It was filtered, then concentrated affording a *yellow* solid **3** (0.141 g, 73.9 %), m.p. 125 °C.

(Found: C, 45.6; H, 4.8; N, 4.45 %; $C_{36}H_{47}N_3O_9PdSCl_2P_2$ requires: C, 46.2; H, 5.0; N, 4.5 %); IR data, Table 1; $^{31}$P, $^1$H and $^{13}$C NMR data, Tables 2, 3 and 4; *m/z* 811 {[Pd(dppe)(GSH)]$^+$ + 1}; tlc, $R_f$ = 0.08 for $H_2O$ solution)

**Synthesis of [Pd(dadpe)(GS.HCl)Cl].3$H_2O$ (4)**

To a suspension of [Pd(dadpe)$Cl_2$].2$CHCl_3$ (0.209 g, 0.245 mmol) in aq. EtOH (30 mL, 1:2) was added GSH (O.074 g, 0.241 mmole) in 10 mL $H_2O$. After stirring at room temperature for 1 h, the resultant clear yellow solution was filtered and the filtrate concentrated affording an orange-yellow solid **4** (0.137 g, 58 %), m.p. 155-160 °C, (Found: C, 43.3; H, 4.6; N, 4.3; Cl, 7.1; P, 3.4 %; $C_{36}H_{47}N_3O_9PdSCl_2PAs$ requires: C, 44.1; H, 4.8; N, 4.3; Cl, 7.2; P, 3.2 %); IR data, Table 1; $^{31}$P and $^{13}$C NMR data, Tables 2 and 4; *m/z* 855 {[Pd(dadpe)(GSH)]$^+$ + 1}; tlc :$R_f$ = 0.15 for $H_2O$ solution (streaking).

**Synthesis of [Pd(dadpe)(N-AcCys)Cl]. $H_2O$ (5)**

To a suspension of [Pd(dadpe)$Cl_2$].2$CHCl_3$ (0.10 g, 0.116 mmol) in EtOH (25 mL) was added an aqueous solution (3 mL) of N-Ac Cys (0.019 g, 0.116 mmol). After stirring at room temperature for 30 min, the solution had become almost clear yellow. It was filtered, then concentrated *in vacuo* affording a *yellow* solid **5** (0.053g, 60.8%), m.p. 145-150 °C.

(Found, C, 48.6; H, 4.7; N, 1.9; P, 4.3 %. $C_{31}H_{34}NO_4SPdPAsCl$ requires: C, 48.7; H, 4.5; N, 1.8;

P, 4.1 %); $^{31}P$ NMR data, Table 2.

Reactions of $[Pd(dppe)Cl_2].2CHCl_3$ with NAcCysH were also carried out, but it was not possible to isolate a pure product.

**Biological testing**

The cytotoxicities of aqueous solutions of the glutathione complexes **3** and **4** against murine L1210 and ADJ/PC6, and normal CH1 (human ovarian cells) cell-lines were determined as described previously.[2]

**RESULTS AND DISCUSSION**

There are a few previous reports of Pd(II) complexes with anticancer activity.[17,18] In general Pd(II) complexes are much more kinetically labile than those of Pt(II) and therefore less likely to arrive at the target site (e.g. DNA) with the original ligands still bound. However in the present case intracellular ligand release might still result in activity since the ligands dppe and dadpe are themselves cytotoxic.[2] The main aim of the present work was to increase the aqueous solubility of Pd(II) complexes containing these hydrophobic ligands whilst retaining their cytotoxicity.

We prepared the novel complexes [Pd(L-L)(Cys)Cl] (L-L′ = dppe **1**; dadpe **2**), [Pd(L-L′)(GS)Cl] (L-L′ = dppe **3**; dadpe **4**) and [Pd(L-L)(N-AcCys)Cl] (L-L′ = dadpe **5**) by addition of an aqueous solution of the thiol ligand to an aqueous ethanolic suspension of $[Pd(L\text{-}L)Cl_2]$. The complexes have satisfactory element analyses, except for the low C content of complex **1**. The mass spectra of complexes **1** to **4** were consistent with the proposed formulae and the presence of monomeric structures in the solid state. Only for complex **2**, [Pd(dadpe)(Cys.HCl)Cl], was a significant amount of a higher molecular mass ion observed, and this was only a minor

**TABLE 1.** IR data for [Pd(L-L′)(Cys.HCl)Cl] (L-L′ = dppe **1**; dadpe **2**) and [Pd(L-L′)(GS.HCl)Cl] (L–L′ = dppe **3**; dadpe **4**) and the thiol ligands. (vs very strong, s strong, m medium, w weak, br broad)

| CysH | 1 | 2 | GSH | 3 | 4 | Assignment |
|---|---|---|---|---|---|---|
| 3460 (m,br) | 3430 (m,br) | 3450 (m,br) | | 3400 (w,br) | 3400 (w,br) | ν(O-H) of $H_2O$ |
| | | | 3330 vs | | | |
| 3180 vs | | | 3240 vs | | | ν(N-H) |
| 3000 (s, br) | 3060 (m, br) | 3050 w | 3120 s | -[1] | -[1] | |
| 2550 m | absent | absent | 2515 s | absent | absent | ν(S-H) |
| - | 1725 (s, br) | 1725 (s, br) | 1710 vs | 1720 m | 1715 (m, br) | ν(C=O) |
| 1600 (vs, br) | 1635 (vs, br) | 1625 (m, br) | 1650 vs | 1650 (s, br) | 1650 (m, br) | $\delta_d(NH_3^+)$ |
| 1580 (vs, br) | absent | absent | 1600 (s, br) | absent | absent | $\nu(COO^-)_{asym}$ |
| 1515 s | 1480 s | 1470 m | 1525 (s, br) | 1515 (w, br) | 1510 (w, br) | $\delta(NH_3^+)_{sym}$ |
| 1415 s | 1430 s | 1425 s | 1405 m | -[1] | -[1] | $\nu(COO^-)_{sym}$ / ν(C-O)/ δ(O-H) in-plane |
| 940 s | 930 (w, br) | absent | 920 s | absent | absent | δ(S-H)/δ(O-H) out-of-plane |

[1] Overlap with Nujol component. The mass spectrum of **1** showed a parent molecular ion (p.m.i) of m/e 624 corresponding to $[Pd(dppe)(Cys)]^+$ which is also the most intense or base peak. For **2** the base peak was at m/z = 670, which is assigned to $[Pd(dadpe)(CysH)]^+$ +1; there were also a number of higher mass peaks, the most abundant being m/z = 1261 ($[Pd(dadpe)(CysH)]_2^+$ - Ph +1; 20% intensity of base peak). Complexes **3** and **4** showed p.m.i.'s at m/z = 811 and 855 corresponding to $[Pd(dppe)(GSH)]^+$ + 1 and $[Pd(dadpe)(GSH)]^+$ + 1, respectively. The base peaks for these two complexes are assignable to a species $[Pd(L-L)(SH_2)]^+$ + 1. The IR spectra for complexes **1-4**, Table 1, showed the disappearance of the SH stretch confirming that the thiol ligands are coordinated via S.

**TABLE 2.** $^{31}P\{^1H\}$ NMR data for [Pd(L-L′)(Cys.HCl)Cl], and [Pd(L-L′)(GSH.HCl)Cl] in $D_2O$ at various pH* values and in $d_6$-dmso, and for [Pd(dadpe)(NAc-Cys)Cl] **5** in EtOH/$D_2O$. (L-L′ = dppe, dadpe) and some tentative assignments.

| Complex | Solvent | pH* | δ (ppm) | J (Hz) | Group *trans* to P |
|---|---|---|---|---|---|
| [Pd(dppe)(Cys)Cl] **1** | $D_2O$ | 2.7 | 65.4 | - | S bridging |
| | | | 64.2 | 18.6 | Cl/N |
| | | | 55.7 | 18.7 | S |
| | | 7.1 | 63.3 | 19.0 | Cl/N |
| | | | 54.9 | 19.4 | S |
| | | 12.2 | 63.3 | 19.0 | Cl/N |
| | | | 54.9 | 19.1 | S |
| | $d_6$-dmso | - | 62.6 | 22.9 | Cl/N |
| | | - | 51.0 | 23.0 | S |
| [Pd(dadpe)(Cys)Cl] **2** | $D_2O$ | 2.6 | 68.5 (br) | - | S bridging |
| | | | 64.9 | - | Cl/N |
| | | | 58.7 | - | S |
| | | 7.1 | 64.3 (80%) | - | Cl/N |
| | | | 58.2 (20%) | - | S |
| | | 12.2 | 64.3 (90%) | - | Cl/N |
| | | | 58.0 (10%) | - | S |
| | $d_6$-dmso | | 63.7 | - | Cl/N |
| [Pd(dppe)(GS)Cl] **3** | $D_2O$ | 2.5 | 65.1 | - | S bridging |
| | | 7.1 | 64.8 | - | S bridging |
| | | 12.5 | 61.0, 52.2, 49.5 (50:30:20%) | - | |
| | $d_6$-dmso | - | 66.4 | 11.9 | Cl/N |
| | | | 54.2 | 11.9 | S |
| | | | 67.4, 56.9 (weak) | - | |
| [Pd(dadpe)(GS)Cl] **4** | $D_2O$ | 2.5 | 67.5 | - | Cl/N |
| | | 7.1 | 67.2 | - | Cl/N |
| | | 12.4 | 63.7, 62.9, 62.4 | - | |
| | $d_6$-dmso | | 68.8 (92%) | - | Cl/N |
| | | | 58.8 (8%) | - | S |
| [Pd(dadpe)(NAc-Cys)Cl] **5** | EtOH/$D_2O$ | 67.2, 66.2-64.2 (weak) | | - | |

**TABLE 3.** $^1$H NMR data for the complexes [Pd(dppe)(Cys)Cl] **1** and [Pd(dppe)(GS)Cl] **3** in $D_2O$. (dd doublet of doublets, t triplet, m multiplet, br broad $\Delta\nu_{1/2}$ 40-100 Hz).

| **Complex** | **δ (ppm)** | **δ (ppm)** | **Assignment** |
|---|---|---|---|
| [Pd(dppe)(Cys)Cl] **1** | pH* = 2.7 | pH* = 7.1 | |
| | 7.875, 7.675 | 7.825, 7.733 | $C_6H_5$ of dppe |
| | 7.600, 7.574 m | 7.648, 7.574 m | |
| | 4.224 m | 3.814 m | Cys α-CH |
| | 2.942 m | 2.862 m | Cys β-$CH_2$ |
| | 2.718 m | 2.778, 2.711 | dppe $CH_2$ |
| | | 2.607 m | |
| [Pd(dppe)(GS)Cl] **3** | pH* = 2.5 | pH* = 7.1 | |
| | 7.88, 7.68 | 7.89, 7.64 | $C_6H_5$-of dppe |
| | 7.55, 7.47 | 7.50, 7.41 | |
| | 7.39 br | 7.34 br | |
| | 3.763 q | 3.657 t | Gly-α $CH_2$ |
| | 3.724 dd | 3.570 | Glu-α CH |
| | | 3.388 | |
| | 3.32 br | 3.27 br | Cys-β $CH_2$ |
| | 2.85 br | 2.82 br | Glu-γ $CH_2$ |
| | 2.52 br | 2.443 br | Glu-β $CH_2$ |
| | 2.231, 2.047 m | 2.22, 2.00 m | $CH_2$ of dppe |
| | 1.966 m | | |

In solution the structures of these complexes are potentially complicated since Pd(II) can form strong bonds to $Cl^-$, thiolate S, carboxylate O, and amino or deprotonated amide N atoms. We made some tentative structural assignments from $^{31}$P, $^1$H and $^{13}$C NMR data. $^{31}$P NMR is particularly useful for dppe complexes since the presence of a $^{31}$P-$^{31}$P coupling indicates a complex with non-equivalent coordinated P atoms and the shifts are a guide to the nature of the trans ligand, based on those of previously-reported chloro (δ 64.2 ppm) and S-bound dmsa (δ

**TABLE 4(a).** $^{13}C\{^1H\}$ NMR data for [Pd(L-L′)(GS)Cl] (L-L′ = dppe **3**, dadpe **4**) in $D_2O$ at acidic and neutral pH* values. Δδ = δ(pH* 7.2)- δ(pH* 1.8) and some tentative assignments.

| Complex | | Δδ(complex) | Δδ(GSH) | Assignment |
|---|---|---|---|---|
| [Pd(dppe)(GS)Cl] **3** | | | | |
| pH* 1.8 | pH* 7.2 | | | |
| 176.1 | 176.6 | 0.5 | 0.4 | Glu γ-CONH |
| 175.5 | 178.4 | 2.9 | 3.3 | Gly- αCOOH |
| 175.1 | 176.4 | 1.3 | 1.6 | Glu- αCOOH |
| 173.0 | 172.0 | -0.8 | -1.0 | Cys- αCONH |
| 136.5, 135.8 | 136.6, 135.9, 135.5 | | | dppe Ph |
| 132.7, 132.5 | 132.9, 132.5 (d 6.4 Hz) | | | |
| 129.0, 128.2, 127.3 | 129.2 br, 128.1, 127.3 | | | |
| 56.8 | 56.8 | 0 | 0.1 | Cys- αCH |
| 55.6 | 56.8 | 1.2 | 1.2 | Glu- αCH |
| 43.8 | 46.1 | 2.3 | 2.2 | Gly- $\alpha CH_2$ |
| 35.1 | 35.4 | 0.3 | 0.2 | Cys- $\beta CH_2$ |
| 34.1 | 34.5 | 0.4 | 0.4 | Glu-$\gamma CH_2$ |
| 30.1 d | 30.0 (d 36.7 Hz) | | | $CH_2$-P |
| 28.4 | 29.0 | 0.6 | 0.5 | Glu- $\beta CH_2$ |
| [Pd(dadpe)(GS)Cl] **4** | | | | |
| pH* 1.8 | pH* 7.2 | | | |
| 176.0 | 176.7 | 0.7 | 0.4 | Glu γ-CONH |
| 175.5 | 178.3 | 2.8 | 3.3 | Gly- αCOOH |
| 175.0 | 176.4 | 1.4 | 1.6 | Glu- αCOOH |
| 173.3 | 172.3 | -1.8 | -1.0 | Cys- αCONH |
| 172.9 | 172.0 | -0.9 | | |
| 172.5 | 171.5 | -1.0 | | |
| 136.7, 136.3 | 136.9, 136.8 | | | dadpe Ph |
| 135.6, 135.2 | 136.3, 136.1, 135.9 | | | |
| 56.8 | 56.8 | 0 | 0.1 | Cys- αCH |
| 55.4 | 56.8 | 1.4 | 1.2 | Glu- αCH |
| 43.8 | 46.0 | 2.2 | 2.2 | Gly- $\alpha CH_2$ |
| 36.7 | | | 0.2 | Cys- $\beta CH_2$ |
| 36.5 (d 14.4 Hz) | | | | $CH_2$-As |
| 34.0 | 34.4 | 0.4 | 0.4 | Glu-$\gamma CH_2$ |
| 31.0 (d 27.3 Hz) | | | | $CH_2$-P |
| 28.4 | 29.0 | 0.6 | 0.5 | Glu- $\beta CH_2$ |

**TABLE 4(b).** $^{13}C$ Coordination chemical shifts (ccs) for [Pd(dppe)(GS)Cl] **3** and [Pd(dadpe)(GS)Cl] **4** at pH* 1.8 and 7.2 (upfield shifts are negative)

| Group | Coordination chemical shifts (ppm) Complex 3 | | Complex 4 | |
|---|---|---|---|---|
| | pH* = 1.8 | pH* = 7.2 | pH* = 1.8 | pH* = 7.2 |
| Glu γ-CONH | -1.2 | -1.1 | -1.3 | -1.0 |
| Gly- αCOOH | -0.2 | -0.6 | -0.2 | -0.7 |
| Glu- αCOOH | 0 | -0.3 | -0.1 | -0.3 |
| | | | -1.9 | -2.1 |
| Cys- αCONH | -2.2 | -2.4 | -2.3 | -2.4 |
| | | | -2.7 | -2.9 |
| Cys- αCH | -1.5 | -1.6 | -1.5 | -1.6 |
| Glu- αCH | -0.1 | -0.1 | -0.3 | -0.1 |
| Gly- $\alpha CH_2$ | -0.1 | 0 | -0.1 | -0.1 |
| Cys-$\beta CH_2$ | 7.0 | 7.1 | 8.6 | -[1] |
| Glu- $\gamma CH_2$ | 0.3 | 0.3 | 0.2 | 0.2 |
| Glu- $\beta CH_2$ | 0 | 0.1 | 0 | 0.1 |

[1] uncertain unassignment

54.4 ppm) complexes. However based on the present data alone a distinction between N and Cl binding was not possible.

$^{31}P$ NMR data are given in Table 2. For complex **1** at low pH* two species were present in solution, one of which contained magnetically equivalent P atoms (δ = 65.4; **1a**) and the other (δ = 64.2, 55.7; **1b**) non-equivalent P atoms. The coupling constant of 19 Hz is typical of $^3J$ (PP) values in square-planar complexes.[2] At higher pH* and in $d_6$-dmso only **1b** was present. This suggests that complex **1a** is a symmetrical thiolate S-bridged dimer $[Pd(dppe)(\mu\text{-Cys-}S)]_2$. It seems likely that complex **1b** is present in dmso as [Pd(dppe)(Cys-*S*)Cl] and in aqueous solution **1b** has a chelated Cys ligand as in [Pd(dppe)(Cys-*S,N*)]. At high pH*, the spectrum of **2** showed two resonances at 64.3 and 58.0 ppm. Since there is only one P atom in the dadpe ligand, these are assigned to two isomeric species, the latter to a P *trans* to S by analogy with [Pd(dadpe)(dmsa)] (57.3 ppm)[2], and the former to the predominant isomer containing P *trans* to N or Cl. At low pH* a third peak is present assignable to a species containing bridging S, and the intensity of the peak for the major isomer increased by a factor of ca. 4.

A pure product was not isolated from the reaction of [Pd(dppe)$Cl_2$] with N-acetyl-L-cysteine; the $^{31}P$ NMR spectrum of an aqueous EtOH solution of the crude product showed that only a species containing magnetically equivalent P atoms was present. Complex **5**, [Pd(dadpe)(NAcCys)Cl] gave two resonances at 76.2 ppm and 66.2 ppm in aqueous EtOH.

$^1H$ NMR data for complexes **1** and **3** are listed in Table 3. For complex **1** at low pH* there are resonances at 4.22 and 2.94 ppm due to the α-CH and β-$CH_2$ of coordinated Cys. At neutral pH, these are less shifted with respect to unbound Cys, in particular that of the α-CH, suggesting that either the carboxylate or the amino group is uncoordinated and protonates at low pH. At both pH values, the $^1H$ NMR spectra showed minor signals characteristic of cystine and free Cys which shifted slightly downfield as the pH* was increased. The spectra at low pH* also showed four multiplets at 7.88-7.57 ppm due to the $C_6H_5$ protons of dppe, and a broad multiplet at 2.718 p.p.m. due to the $CH_2$ protons of dppe. At higher pH* the Ph resonances were unchanged and the $CH_2$ protons gave rise to three multiplets. The spectrum of **3** at low pH* showed a quartet assignable to Gly-α-$CH_2$ protons and a doublet of doublets due to Glu-αCH; these were sharp and overlapping. Other resonances assigned to GS in this complex were broad, i.e. those of the Cys-β$CH_2$ protons and of Glu–γ$CH_2$ and Glu-β$CH_2$. No resonance due to Cys-α-CH was observable. At neutral pH* the resonances for Glu-αCH appeared as two doublet of doublets with a shift difference of 93.8 Hz. The resonances for Cys-β$CH_2$ and γ-Glu $CH_2$ were broad at this pH*. Resonances due to protons of $C_6H_5$ groups of dppe were broad at both pH values. The resonances of the $CH_2$ protons of dppe appeared as a set of three multiplets at acidic pH and at higher pH* they gave rise to two multiplets. Added GSH gave rise to a separate set of sharp resonances and so exchange with free GSH was not the cause of the broadening. Instead, this is probably due to the presence of coordination equilibria in solution. Spectra for **2** and **4** were complicated by the presence of two isomeric products, as discussed above, and so are not reported.

$^{13}C$ NMR data for the complexes [Pd(L-L')(GS)Cl] (L-L′ = dppe **3** ; L-L′ = dadpe **4**) at low and neutral pH* values are given in Table 4(a). The assignments for complex **3** are made partly on the basis of a comparison of the changes in shifts of the resonances on increasing the pH* from 1.8 to 7.2 with those observed for free GSH,[19] and on the shifts noted previously for the Cys carbons of a 2:1 solution of GSH and $Hg(NO_3)_2$ in this pH* range.[20] At pH* = 7.2, three sets of broad resonances were observed in the phenyl region corresponding to the $C_6H_5$ carbons of dppe. The spectrum of the glutathione carbons of complex **4** was similar except that there were three resonances in the Cys α-CONH region at δ = 172.5, 172.9, and 173.3 ppm. The resonances at δ = 36.23 and 30.93 ppm are assigned to the aliphatic carbons adjacent to As and P, respectively. The latter resonance is a doublet with $J_{P\text{-}C}$ 27 Hz. There were several resonances in the aromatic region of the spectrum corresponding to $C_6H_5$ of dadpe.

The coordination chemical shifts of the carbon atoms are listed in Table 4(b). The greatest shift observed is for the β-$CH_2$ carbons of Cys which are shifted downfield by 7 to 8.6 p.p.m confirming coordination of the adjacent S atom. In addition the α-CH carbons of Cys show an upfield shift of 1.6 p.p.m.. The resonances assigned to the Cys α-CONH are also shifted upfield (2.2 to 2.4 p.p.m.) as are the resonances of the Glu γ-CONH (1.0 to 1.3 p.p.m.) suggesting interactions between the NH or CO groups and the metal centre. The coordination shifts are independent of pH* with the exception of those of the Glu α-COOH which is higher at pH* = 7.2, perhaps because the coordination of the metal affects the $pK_a$ of the carboxylic acid group. The magnitudes of the coordination shifts for complexes **3** and **4** are similar, suggesting a similar coordination environment in both.

A previous study of Pd(II) GSH complexes suggested coordination via S and N of the Cys residue based on IR, UV-visible and analytical data, as well as the presence of bridging chloride ligands.[4] Another study[3] of a 1:1 complex suggested the formation of a seven-membered ring involving Pd, the terminal amino group and the deprotonated NH of the first peptide bond (Cys) in the case of the 1:1 Pd: GSH. Competition between S, N, Cl and O ligands is therefore likely to be facile on Pd(II) accounting for the mixtures of species that are sometimes observed in solution.

The glutathione complexes, **3** and **4**, [Pd(L-L)(GS.HCl)Cl], were tested for toxicity against three cell-lines: the murine cell-lines L1210 and ADJ/PC6, and the normal human ovarian cell-line CH1. The compounds had a relatively low toxicity against the murine cell-lines, Figure 1A, e.g. towards L1210 the $IC_{50}$ values for **3** and **4** were ca. 22 and 32 μM respectively. However, they showed some tissue selectivity against CH1 cells with $IC_{50}$ values in the region of 4 to 10 μM (cf *cis*platin $IC_{50}$ $10^{-7}$ μM against this cell-line). They were more cytotoxic towards L1210 cells than the *bis*thiolate complexes we have investigated previously, [Pd(dppe)(dmsa)] and [Pd(dadpe)(dmsa)],[2] and of comparable cytotoxicity to the square-planar dichloro complexes, [Pd(L-L')$Cl_2$], as shown in Figure 1B. However there is no increase in the cytotoxicity of the complexed ligand dppe or dadpe with respect to that of the free ligand towards this cell-line. Indeed, for dppe the ligand itself is significantly more toxic (by up to 5 times).

### CONCLUSION

By introducing thiolato ligands into Pd(II) dppe and dadpe complexes, we have improved their aqueous solubility and retained their cytotoxicities in comparison to the parent dichloro complexes. For the L-cysteine complexes **1** and **2**, IR and NMR showed the presence of S-bound cysteine ligands, and there was evidence for both monomers containing S,N chelates and dimers with bridging S in solution. Depending on the conditions, glutathione also bound strongly via S in

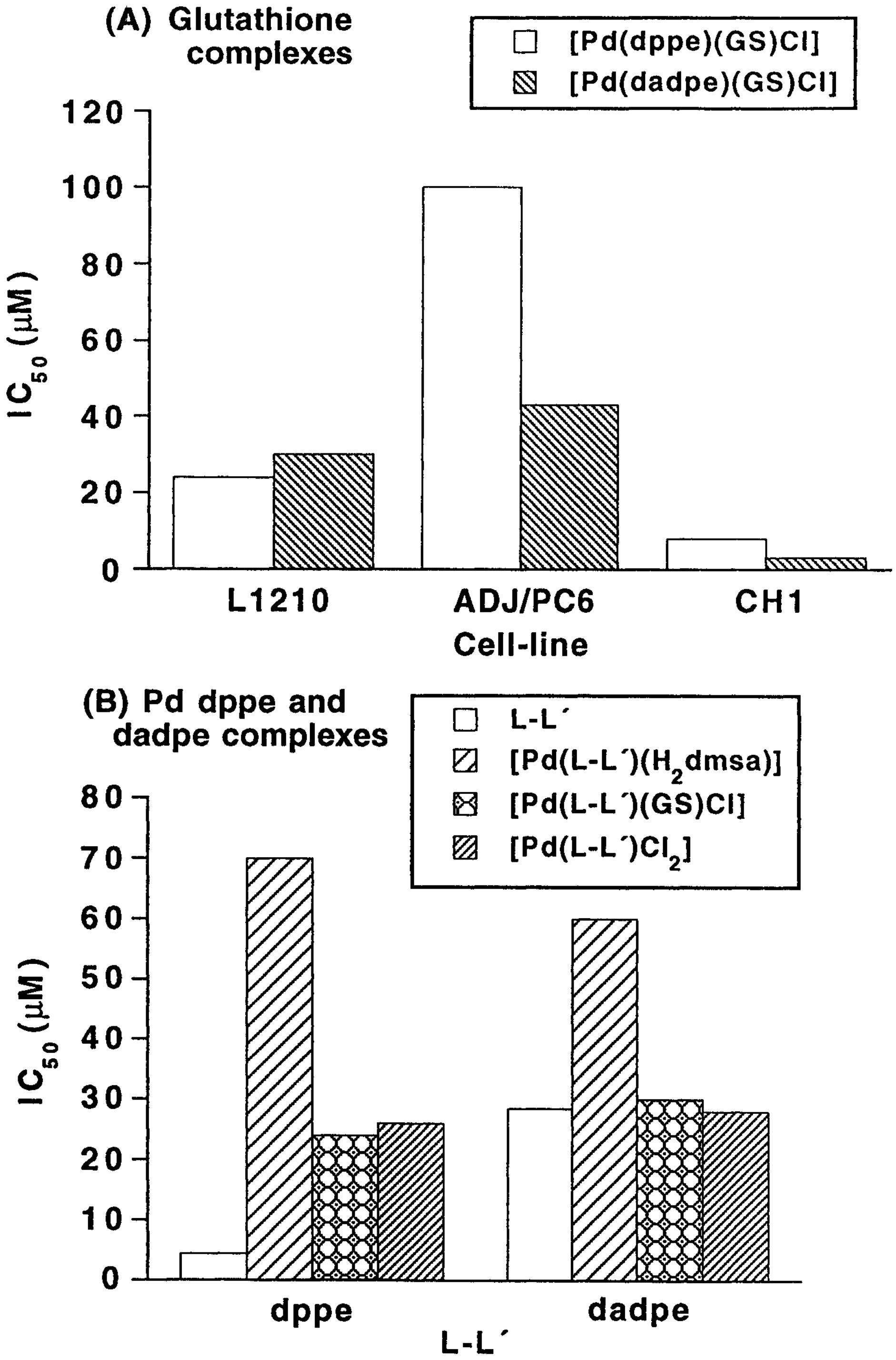

Figure 1.

(A) Cytotoxicities of Pd(II) glutathione complexes towards three cell lines

(B) Cytotoxicities of dppe and dadpe Pd(II) complexes of glutathione (GSH), dimercaptosuccinic acid ($H_2$dmsa) towards L1210 cells compared to those of the free ligands dppe, dadpe, and their Pd(II) dichloro complexes.

complexes **3** and **4** and S-bridged complexes appeared to predominate in aqueous solution, but complicated equilibria were also detected with evidence for exchange between various coordination sites. Complexes with moderate cytotoxicity such as those described here, if they are also active *in vivo*, could be useful in combination therapy with established drugs such as cisplatin since they are likely to have a different mechanism of action.

### ACKNOWLEDGEMENTS

We thank the Cancer Research Campaign, the Medical Research Council, University of London Intercollegiate Research Services and the Wolfson Foundation for their support for this work. We also thank Prof. K. Harrap and Dr. P. Goddard, Institute of Cancer Research, Sutton for carrying out the cytotoxicity experiments. One of us (E.G.F.) thanks the University of Santiago de Compostela and Xunta de Galicia, Spain for financial support.

### REFERENCES

1. S.J. Berners-Price and P.J. Sadler, *Struct. Bonding* **70**, 27 (1988)
2. O.M. Ni Dhubhghaill, P.S. Jarrett and P.J. Sadler, *J. Chem.Soc. Dalton Trans.* 1863 (1993)
3. L.D. Pettit and M. Bezer, *Coord. Chem. Rev.*. **61**, 97 (1985)
4. S.T. Chow, C.A. McAuliffe, and B.J. Sayle, *J. Inorg. Nucl. Chem.* **37**, 451 (1975)
5. G.D. Zegzhda and T.V. Zegzhda, *Russ. J. Inorg. Chem.*. **23**, 1826 (1978)
6. D. L. Rabenstein, S. J. Backs, A.A. Isab, *J. Am. Chem. Soc.* **103**, 2836 (1981)
7. W. Kadima and Dallas L. Rabenstein, *J. Inorg. Biochem.* **38**, 277 (1990)
8. M.T. Razi, G. Otiko, and P.J. Sadler in *Platinum, gold and other metal chemotherapeutic agents, ACS Sym. Ser.* **209**, 371 (1983).
9. D. L. Rabenstein and M.T. Fairhurst, *J. Am. Chem. Soc.* **97**, 2086 (1975)

10. R.S. Reid and D.L. Rabenstein, *Can. J. Chem.* **59**, 1505 (1981);
R.S. Reid and D.L. Rabenstein, *J. Am. Chem. Soc.* **104**, 6733 (1982)

11. D.L. Rabenstein and R.S. Reid, *Inorg. Chem.* **23**, 1246 (1984);
B. V. Cheesman, A. P. Arnold, and D. L. Rabenstein, *J. Am. Chem. Soc.* **110**, 6359 (1988)

12. K. Miyoshi, Y. Sugiura, K. Ishizu, Y. Iitaka, and H. Nakamura, *J. Am. Chem. Soc.* **102**, 6130 (1980)

13. L.P. Battaglia, A. Bonamartini Corradi, C. Grasselli Palmieri, M. Nardelli, and M.E. Vidoni Tani, *Acta Cryst.* **B29**, 762 (1973)

14. A. Allain, M. Kubiak, B. J. Jezowska-Trzebiatowska, H. Kozlowski, and T. Glowiak, *Inorg. Chim. Acta* **46**, 127 (1980)

15. M. Kubiak, A. Allain, B. J. Jezowska-Trzebiatowska, T. Glowiak, and H. Kozlowski, *Acta Cryst.* **B36**, 2246 (1980)

16. G.K. Anderson, H.C. Clark, and J.A. Davies, *Inorg. Chem.* **20**, 3607 (1981)

17. D.S. Gill, in *Platinum Coordination Complexes in Cancer Chemotherapy*, M.P. Hacker, E.B. Douple, I.H. Krakoff, Eds., Martinus Nijhoff, Boston, p 267 (1984).

18. P. Castan, E. Colacio-Rodriguez, A.L. Beauchamp, S. Cros and S. Wimmer, *J. Inorg. Biochem.* **38**, 225 (1990)

19. G. Jung, E. Breitmaier, and W. Voelter, *Eur. J. Biochem.* **24**, 438 (1972)

20. B.J. Fuhr and D.L. Rabenstein, *J. Amer. Chem. Soc.* **95**, 6944 (1973)

**Received: June 6, 1994 - Accepted: June 23, 1994 - Received in camera-ready format: September 15, 1994**

